# Patient safety during joint replacement surgery: experiences of operating room nurses

**DOI:** 10.1136/bmjoq-2021-001604

**Published:** 2021-11-07

**Authors:** Anette Nyberg, Birgitta Olofsson, Volker Otten, Michael Haney, Ann-Mari Fagerdahl

**Affiliations:** 1 Department of Nursing, Faculty of Medicine, Umeå University, Umeå, Sweden; 2 Department of Surgical and Perioperative Sciences, Faculty of Medicine, Umeå University, Umeå, Sweden; 3 Department of Surgical and Perioperative Sciences, Orthopedics, Faculty of Medicine, Umeå University, Umeå, Sweden; 4 Department of Surgical and Perioperative Sciences, Anesthesiology, Faculty of Medicine, Umeå University, Umeå, Sweden; 5 Department of Clinical Science and Education, Södersjukhuset, Karolinska Institutet, Stockholm, Sweden

**Keywords:** management, patient safety, attitudes

## Abstract

**Background:**

Avoidable complications for surgical patients still occur despite efforts to improve patient safety processes in operating rooms. Analysis of experiences of operating room nurses can contribute to better understanding of perioperative processes and flow, and why avoidable complications still occur.

**Aim:**

To explore aspects of patient safety practice during joint replacement surgery through assessment of operating room nurse experiences.

**Method:**

A qualitative design using semistructured interviews with 21 operating room nurses currently involved in joint replacement surgery in Sweden. Inductive qualitative content analysis was used.

**Results:**

The operating room nurses described experiences with patient safety hazards on an organisational, team and individual level. Uncertainties concerning a reliable plan for the procedure and functional reporting, as well as documentation practices, were identified as important. Teamwork and collaboration were described as crucial at the team level, including being respected as valuable, having shared goals and common expectations. On the individual level, professional knowledge, skills and experience were needed to make corrective steps.

**Conclusion:**

The conditions to support patient safety, or limit complication risk, during joint replacement surgery continue to be at times inconsistent, and require steady performance attention. Operating room nurses make adjustments to help solve problems as they arise, where there are obvious risks for patient complications. The organisational patient safety management process still seems to allow deviation from established practice standards at times, and relies on individual-based corrective measures at the ‘bedside’ at times for good results.

## Introduction

Perioperative and postoperative complications result in much patient morbidity and mortality,^1 2^ and some of these are preventable.^3 4^ Often, well-founded treatment recommendations are available to help to prevent complications. Implementation and maintenance of evidence-based practice is a challenge, including in perioperative practice where there are complex organisational challenges. Good technical and non-technical skills are needed in order to reduce and manage threats to patient safety.^5^ One example of this is the WHO Surgical Safety Checklist (SSC),^6^ which was launched in 2008. Employing the SSC is an example of consolidating individual, team, and organisational planning and control steps designed to reduce the risk of avoidable complications for patients (enhance patient safety).

Joint replacement surgery is a complex but largely standardised operation where surgical complications can be devastating for patients. Besides optimising operative logistics, all available steps to minimise risk for surgical site infection (SSI) or periprosthetic joint infection (PJI) are incorporated into current arthroplasty operations, given that PJIs are recognised as devastating for patients where they occur.^7^ Operating room nurses (ORNs) are constantly managing risks and preventing harm with the aim of zero perioperative mishaps and zero postoperative infections.^8^ Nevertheless, they occur with alarming frequency.^4 9^ Deviations from widely accepted best practice occur, but it is unclear to what extent that these will increase risk for complications. Still, to work to minimise risk means to find the practical steps in practice where perioperative staff experience that commonly accepted or established good practice^10–12^ is difficult follow.

This study aimed to explore current experience of ORNs focused on their routine workplace reflections concerning patient safety factors that they can influence. The specific aims were to present and analyse current experience for ORNs in their work to prevent perioperative mishaps and SSI risk for a very frequently done operation, where individual, team and organisation factors are relevant.

## Method

### Design

This was a qualitative study analysing data collected through semistructured interviews. The reporting complies with Consolidated Criteria for Reporting Qualitative Research.^13^

### Setting and Swedish context

The interviews were conducted at three different hospitals in Sweden: one university hospital, one public general hospital and one private orthopaedic hospital.

There are international differences concerning areas of responsibility in the OR. In Sweden, ORNs are responsible for preparing instruments and implants for the operation, patient positioning and OR asepsis.^14^ The ORN training in Sweden starts as a registered nurse, then a 1-year ORN specialist programme, with certification and a master’s degree.^14–16^

### Participants

Inclusion criteria were: certified and active ORNs, with at least 1 year of experience of joint replacement surgery. Purposive sampling was performed. Nursing department leaders identified ORNs who met inclusion criteria. Twenty-one ORNs were interviewed, median age 43 years (range 27–64), 2 men and 19 women. Median experience as ORN was 7 years (range 2–41).

### Data collection

A semistructured interview guide consisted of three questions: ‘What does patient safety in perioperative care during arthroplasties include for you?’, ‘What do you consider most important in securing patient safety?’ and ‘Do you detect any weak areas in patient safety?’. Probing questions were asked when needed. Data were collected from April 2020 to August 2020. All interviews were conducted by the first author. Sixteen of the 21 interviews took place in person, and 5 by telephone. The interviews lasted median 31 min (range 20–45) and were digitally recorded and transcribed verbatim.

### Data analysis

The responses were analysed inductively using qualitative content analysis, which is used to describe similarities and differences in the manifest content and to interpret the latent content in the phenomenon under exploration.^17–19^ We performed a manifest analysis. The transcripts were imported into MAXQDA 2020 (VERBI Software, Germany). First, the recorded interviews were reviewed and transcripts read through to get a sense of the whole. Second, the text was divided into meaning units relevant to the aim of the study. Third, the meaning units were carefully condensed to avoid losing content, and each meaning unit was coded. The codes were abstracted into subcategories and categories. The analysis was discussed among coauthors until consensus was reached.

## Findings

From the analysis, three main categories and seven subcategories emerged (table 1).

**Table 1:** Categories and subcategories

Categories	Subcategories
Organisational level	Reliable procedural plan Functional reporting and documentation
Team level	Interprofessional and interdisciplinary collaboration Protocol, checklist and standardisation implementation Aseptic principles compliance
Individual level	Professional knowledge, skills and experiences Personal engagement

### Organisational level

Important preconditions on the organisational level that were highlighted included a reliable plan for the procedure, and reporting and documentation practices enabling a functional exchange of information.

#### Reliable procedural plan

The ORNs stated their need for a reliable preoperative plan to ensure a safe procedure. By planning and preparing well for the procedure, they attempted to reduce the time for surgical procedure. Before preparing for the procedure, they often needed to confirm the information from a computerised surgical planning system with the orthopaedic surgeon, due to occurrence of failure in updating the plan. This need to confirm the plan was perceived as unsatisfying and experienced as time-consuming. Though instead of addressing the main problem, the participants resorted to adapting to the incomplete planning.

But it requires extra work when something does not seem to be right, that is when you start to suspect something, that someone has to call and talk to those who are going to operate and so extra time and energy goes into something that should not be needed. (IP16)

Ensuring the availability of instruments and implants for the planned procedure was considered a challenge. With procedural planning not being updated, essential instruments or implants for the procedure could be missing, and the ORN needed to start preparing for another surgical system instead. This was considered a disturbance in workflow in the OR, and made it difficult to accomplish the list of operations scheduled for the day. The participants needed to prepare fast-paced, and expressed concern for missing crucial items when doing so.

For some participants, the main source of patient-related information was derived from the surgical planning system and the anaesthesia preoperative assessment. They did not find time to get information from the main health records, and were thereby not routinely accessing information which included notifications from orthopaedic ward nurses, such as already-identified patient risk for pressure ulcers and nutrition status. Therefore, the possibility to prepare for a safe procedure was reduced, and they experienced important patient information in different computerised systems in parallel a risk.

#### Functional reporting and documentation

There were concerns that the reporting and documentation practices threatened continuity of patient care. Some participants explained that they were documenting their perioperative care both in the computerised system and also in paper form. This practice was considered both time-consuming and a risk for patient safety. The documentation on paper was deemed necessary as ORNs rarely reported in person to colleagues on the postoperative ward. The nurses on the postoperative and orthopaedic ward were working in the main health record system, not in the planning system where ORNs documented their care, and thereby could not consult the information the ORNs had documented there. The participants suggested that the planning system should be seen merely as a planning system and not as a tool for documentation.

A big problem (documentation) and above all, you are not used to each other’s journal system so the nurses at the postoperative wards rarely go in and read. So, if we write: See Orbit (the planning system), they don’t care about it because they think it is hard to get into it because it is not a system they usually work within. So, all that is important, you have to either say it or write it on the paper and then it becomes double documentation, which feels unnecessary time consuming when we now have so little time on everything as it is. (IP7)

Reporting to a colleague and at the same time keeping the surgery on course was perceived as a risk. ORNs tried to avoid personnel breaks or shift changes during surgery, but sometimes it was seen as inevitable, especially during revision joint replacement surgery lasting the whole day. It was considered a challenge to remember to report everything, and some recalled the need to call the OR on their way home to fill in missing parts in their report.

The participants in this study were not convinced that reporting incidents led to any actual improvements in patient safety. OR management sent information about new routines and incidents by email, and these were sometimes perceived to not reach the appropriate OR personnel. The ORNs voiced a desire to receive feedback on the treatment results for the patients. They wanted to know if there had been injuries for patient were there had been intraoperative challenges. If the orthopaedic surgeons were asked for the results for one specific patient, they shared the result with the ORNs, but there was no systematic feedback on results or complications. For example, some participants emphasised that they wanted to know the infection rates for their specific department.

### Team level

Collaboration, established safety controls and compliance with aseptic principles were stated as important aspects for safety practice within the team. Compliance with aseptic principles was considered to vary among different professions within the team.

#### Interprofessional and interdisciplinary collaboration

The importance of teamwork and collaboration for patient safety was mentioned by all participants. The team shared a goal to do the best for each patient, and every professional’s expertise was a valuable contribution. The ORNs were expecting all team members to perform responsibly, and when this was not the case, it became a strain in the workplace for them. In this collaboration the ORNs felt that their professional knowledge was respected. Steady communication with other OR personnel was seen important.

A lot of teamwork, because I mean, without my anesthesiologist, without my anesthesia nurse, without my assistant nurse, without my surgeon, without me there will be no surgery and without the patient there neither be any surgery. So, there are many people and it gives a sense of security in fact that everyone tries to think; Avoid injuries, not to cause harm - how will it be best. (IP1)

The work required that the ORNs constantly develop their skills, and they tried to improve their work steadily. Being alone in their profession as ORN in the OR put limits on opportunities to ask colleagues for advice and support. In situations where two ORNs collaborated during surgery, they had opportunities to support and learn from each other, and thereby improve their work.

#### Protocol, checklist and standardisation implementation

The participants explained that they were responsible for several established safety controls, including to ensure that all instruments and other sterile material needed were available and functioning—surgical count. The first surgical count was performed before the patient entered the OR. Another surgical count was performed during the surgical procedure before closing the wound to ensure that no surgical items were accidentally left in the operative site.

Another important responsibility for the ORNs was to verify the patient identity when entering the OR, as well as confirming the indicated side on the patient with the planning and the x-rays. Through the timeout in the SSC, the participants confirmed the preparation with the orthopaedic surgeon. However, they experienced that the SSC was not always implemented as designed. The checklist was often performed while the ORNs were busy with final preparations, and not participating with whole attention. How the timeout was performed depended on the orthopaedic surgeon’s interest in the checklist, and differed from one surgeon to another.

… that I know that I have the right patient to begin with, that planning is consistent with the x-rays or when we do ‘Sign in’ that it is consistent with the patient records and the ID bracelet and side, that you operate on the correct side (IP11)

The ORNs considered established safety protocols necessary. For example, using a protocol to ensure availability for the correct implant was named as important. This step is performed by both the orthopaedic surgeon and the ORN, reading on the implant package before it was opened and delivered to the sterile field. Some participants considered standardisation of procedures as a way to ensure safety. They explained that they were constantly assessing what was best for each patient, emanating from existing routines and standardisation.

#### Aseptic principles compliance

The ORNs stated being responsible for sterility and infection control was within the domain of their main concern. They kept guarding sterility throughout the entire surgical procedure by keeping an eye on the activities of other team members, which sometimes could be challenging.

Participants noted that the prerequisites for work in an aseptic environment were present. National guidelines for preventing PJIs were established, and there was most often compliance with these. One example given was a guideline to control the traffic and avoid disturbance of the ventilation by opening the doors and trying to minimise the number of persons in the OR. Still, some participants experienced that compliance with guidelines varied within the team. For example, some orthopaedic surgeons followed the guidelines more strictly than others. With an interesting surgical case, minimising the amount of personnel in the OR was disregarded by some surgeons.

The ORNs also emphasised a need to improve staff behaviour regarding adherence to the aseptic principles. When they insisted on observing aseptic protocols, it sometimes was considered a disturbance of the flow, affecting both the surgical procedure and the whole day operation schedule. Such protocols could be handling of specimens and urinary tract catheters. When notifying others on breaks of aseptic principles, some participants perceived that they were seen as annoying.

So it is, we are herd animals, we do as our colleagues do and it is uncomfortable if someone gives a reprimand or is a hygiene-witch… Even if people might think you're irritating, I think you still get some kind of respect in that you have competence and can see that this is important. Even if you are considered awkward, you are trusted as the person who also is competent and good, good for the group and for the patient.

(IP10)

### Individual level

The ORNs felt a personal responsibility to the patients, and felt guilty when failing to protect the patient from harm. They protected patients by using their professional knowledge and skills, and by having the confidence to speak up if the patient was put at risk in the process.

#### Professional knowledge, skills and experience

The ORNs knew what was expected of them to preserve patient safety. They used professional knowledge and skills to protect patients from risks. This included positioning the patient safely on the operating table among others. They had a responsibility for what they saw during the procedure by being a part of the operating team. If a situation occurred putting the patient at risk, the ORNs had to speak up. They experienced that notifying the orthopaedic surgeon of near misses required confidence, and this was gained through experience. Unexpected things happen during surgery, and the participants needed to be prepared to adjust their plan.

If you see something in the surgical field, I feel that you have a responsibility when you see something, then of course it depends on experience, how much experience you have and what you know and what you have seen before, but it is also a part of patient safety that you are involved in the operation and are taking part in the operation. (IP17)

#### Personal engagement

ORNs felt a great responsibility for the patients’ sense of security and comfort in perioperative care. They had the ambition to greet the patient before starting preparation and wanted patients to know that everyone in the OR would do their best for them in a dignified way.

The participants perceived a feeling of guilt when situations occurred and they failed to protect the patient from coming to harm. Some described situations occurring where they would not have wanted to be the patient. Although the ORNs were well aware that many factors could have led to an infection, they felt accountable for it if a patient acquired an SSI or a PJI.

Then you are as an OR nurse, there must be something that we have in us, as soon as there is an infection then you have to go straight into the old medical record and see if you were there during the previous operation. Because you think that you, yourself carry the responsibility, and of course I understand that it is not how it is, there are many factors that come into play, but I think many of us feel responsible; have I done something wrong, is it my mistake now that led to this patient becoming infected? (IP8)

## Discussion

These findings show that ORNs identify several measures at different levels (organisation, team and individual) where there can be safety improvements in the OR during joint replacement surgery. These occurrences cause disruptions in the workflow and sometimes even threatening patient safety. These findings also show how upcoming problems that occur were solved in everyday work, to get on with the job and to maintain safety.

To ensure a safe surgical procedure, the ORNs identified a need for a reliable preoperative plan, which confirms previous findings.^20^ This is in line with resilience engineering, where predictive information is needed to anticipate for decision making.^21^ Some participants identified problems when confirming the plan between the computerised planning system and the surgeon, which was considered time-consuming and unsafe. The purpose of computerised planning systems is to organise a reliable plan^22^ but achieving this requires an engagement of all stakeholders involved in the planning.^23^ Without an updated plan there is a risk for disruptions which can both compromise patient safety and affect productivity of the organisation, similar findings are presented in previous reports.^22 24^ Participants gave priority to fixing the immediate problem in order to maintain the workflow in OR, instead of addressing the main issue, that the plan was not adequately updated. This can be seen as resilience by the frontline workers,^25^ where the ORNs adapted in order to accomplish a good outcome even when their working conditions were disadvantageous. Although the intention of the ORNs adaptability is good, it can create a disconnection between the OR management’s work-as-imagined and the ORNs’ work-as-done. This type of disconnection can constrain the possibility for necessary change. Work-as-imagined means practising by following protocols and standards for safe performance,^25^ but the staff in OR know that a certain performance variability is expected, as is practice safely. In this study, the underlying problem with low engagement in planning documentation can continue as long as the OR managers do not recognise the problem or do not have the opportunity to solve it. The issue with the planning documentation not being updated might be considered as an annoying repetitive problem by ORNs. In the long term, repetitive annoying problems that lead to frustration, cynicism and turnover among the front-line staff impact the resilience in the system.^21^

Diverse computerised systems were seen obstructing the transfer of information, with a potential to affect the care of patients, which confirms previous findings on documentation.^26^ The ORNs did not find time to access information from the main health record. A technology allowing fast access to relevant information should be provided by the organisations.^24^ In this study, the ORNs did not report directly, or in person, to the postoperative ward, and this to save time. To avoid gaps in the continuity of care which could result, some reported developing a workaround in their documentation practice. This workaround necessitated duplication of documentation in separate systems to ensure that the nurses on postoperative ward received relevant information. Participants adapted their routines, demonstrating resilience to avoid system miscommunication. This development of workarounds can involve taking a risk as well as limiting the possibility for a change.^21^ The risk involved documenting in two places and continuing working in diverse computerised systems alleviated pressure on the hospital management for process improvement. Documentation practices may be directed by traditions and everyday conditions but to ensure safe care the documentation tool should align with the actual practice.^27^ Some suggested that the planning system should be only a tool for planning, not for documentation, and that documentation should be managed in one patient record system.

It was experienced that teamwork and collaboration are crucial in preventing adverse events, which supports findings of previous studies.^28 29^ Expertise from every profession was considered a valuable contribution to the process and every team members need to feel confident to speak up when alert is needed, as reported previously.^30^ The ORNs felt respected for their professional knowledge in collaboration with other team members, although previous research suggests that the collaboration between nurses and physicians needs improvement, both in OR^31^ and in other context within the hospital.^32^ Their professional skills and experience informed their patient safety practice. A combination of technical skills and care for the patient were identified as important, similar findings have been presented earlier.^20 33^ A sense of personal engagement among the ORNs was prominent. Guilty feelings were named if they failed to protect a patient from harm. They also expected all team members to accept their professional responsibilities and emphasised need for a shared goal. An open dialogue within the team, with confirmed expectations were noted as important, which confirms a previous report.^34^ The ORNs identified primary responsibility in assuring compliance with aseptic principles. They experienced variable compliance with this within the team, as has been reported previously.^35^ Guidelines for preventing infections were established, but the experience was that the levels of interest to comply varied among team members. For best effect, teams should share goals and accept workgroup hierarchy, which includes mutual respect and good leadership within the team.^36^ Where some everyday performance variability is expected, things should still go right. Individual performance variability should not be understood as necessarily dangerous, though a team not complying with existing safety protocols is beyond expected performance variability.^37^ Patient safety cannot be improved solely by introducing safety policies—they need to be implemented.^38^

Safety controls, such as checklists, were identified as crucial for preserving safety during surgery, as presented in an earlier report.^39^ Control steps were seen as guidance for safe practice, and specifically SSC as a useful tool to maintain a high level of safety awareness. In other reported findings SSC was noted to increase communication and teamwork in OR^40 41^ and to recognise potential risks.^42^ In this study, it was noted that SSC was not always used as designed mostly dependent on the surgeon. Perceptions of the SSC as nonessential complicated the implementation of SSC.^43^ Previous reporting suggests that the implementation of SSC might be more successful where physicians engaged with leading it.^44^ Standardisation of procedures was considered a way to improve the perioperative process. Both appropriate standardisation and certain flexibility are needed for an organisation to succeed.^45^ While standardisation protects against predictable and preventable errors, flexibility supports resilience in unpredictable situations, where balance between these is needed.^46^ This is in line with another report that suggests that to manage the complexity in OR and maintain safe care necessitates the ability to respond to both the expected and the unexpected.^39^ Our findings show that resilience exists within the organisations, but it is not only used in unpredictable situations. The ORNs demonstrated resilience in managing the everyday work. This shows ORNs ability to make adjustments and maintain them. However, where there is every day need for resilience, capacity to respond to new challenges may be restricted.^21^ Resilience should be needed for resolving unplanned situations rather than everyday occurrences. Safety threats in everyday work should be recognised and managed as the organisation improves.

### Strengths and limitations

During the analysis, there were interactive discussions within the research group which increases credibility for this study. Direct quotations with descriptions are presented for credibility. The diversity of professionals involved in the analysis also strengthens this study. The degree of transferability must be up to the reader.^18^ One limitation is that all interviews were conducted by the first author, who is an ORN and professionally known to some of the participants. Dual roles for interviewer can potentially influence interviewee reporting.^47^ This dual interviewer role may have helped create a safe environment, and the shared understanding can deepen the reporting.^48^

## Conclusion

The conditions to support patient safety, or limit complication risk, during joint replacement surgery continue to be at times inconsistent, and require steady performance attention. ORNs make adjustments to help solve problems as they arise, where there are obvious risks for patient complications. The organisational patient safety management process still seems to allow deviation from established practice standards at times, and relies on individual-based corrective measures at the ‘bedside’ at times for good results.

## Data Availability

Data can be made available on reasonable request.
